# Long-Term Selection with α-Solanine Enhances Tolerance and Drives Multilevel Physiological Remodeling in *Spodoptera frugiperda* (J.E. Smith) (Lepidoptera: Noctuidae)

**DOI:** 10.3390/insects17090941

**Published:** 2026-09-08

**Authors:** Yi-Kuan Wu, Peng-Fei Zhang, Jia-Qi Wu, Bo Pang, Guo-Qing Li, Lin Jin

**Affiliations:** 1 State Key Laboratory of Agricultural and Forestry Biosecurity, College of Plant Protection, Nanjing Agricultural University, Nanjing 210095, China; 2023202051@stu.njau.edu.cn (Y.-K.W.); 2020102079@stu.njau.edu.cn (P.-F.Z.); 2026002008@stu.njau.edu.cn (J.-Q.W.); ligq@njau.edu.cn (G.-Q.L.); 2 Institute of Agriculture, Xizang Academy of Agricultural and Animal Husbandry Sciences, Lasa 850000, China; 13989010631@163.com

**Keywords:** fall armyworm, α-solanine, host adaptation, detoxification, metabolomics, transcriptional remodeling

## Abstract

The fall armyworm, *Spodoptera frugiperda* (J.E. Smith) (Lepidoptera: Noctuidae), is a polyphagous pest exposed to plant defensive chemicals. We tested whether sustained selection by the potato steroidal glycoalkaloid α-solanine induces changes in tolerance, life-history performance, and physiology. A potato-sprout-derived preparation was standardized for α-solanine content by targeted LC–ESI–MS/MS, and a selected line was maintained for 12 generations on a diet containing 1.0 mg α-solanine g^−1^. Selected and unselected lines were compared across normal artificial diet, α-solanine diet, and potato foliage using bioassays, life-history analyses, detoxification-enzyme assays, untargeted metabolomics, and RT-qPCR. By generation 12, the selected line reached a tolerance ratio of 3.52. Under α-solanine exposure, selection shortened larval, pupal, adult, and total development and increased pupation, adult emergence, and egg hatch, while pupal mass and fecundity did not differ significantly between lines. The selected line showed faster demographic turnover. Carboxylesterase activity differed between lines, whereas CYP450 and GST responses were diet-dependent. Metabolomic contrasts indicated a reduced feature-level response to α-solanine in the selected line, and RT-qPCR revealed constitutive and diet-dependent shifts in detoxification- and stress-related genes. Thus, sustained α-solanine selection generated coordinated multilevel remodeling that improved performance under α-solanine exposure and altered responses to potato foliage.

## Simple Summary

The fall armyworm feeds on many crops and encounters diverse plant defensive chemicals. We examined whether repeated exposure to α-solanine, a defensive compound associated with potato, changes how this insect performs and responds physiologically. A line maintained for 12 generations on an α-solanine-containing diet became more tolerant to the compound. Under α-solanine exposure, the selected insects developed faster and showed higher pupation, adult emergence, and egg hatch rates, while pupal mass and egg production remained similar to the unselected line. The selected line also showed faster population turnover and distinct patterns of detoxification-enzyme activity, metabolism, and expression of detoxification- and stress-related genes. Its metabolic response to α-solanine was smaller than that of the unselected line, and its response to potato foliage was also altered. These findings show that sustained exposure to one plant defensive compound can produce coordinated changes across survival, development, reproduction, and physiology, helping explain how a pest that feeds on many crops adjusts to challenging plant chemistry.

## 1. Introduction

The fall armyworm, *Spodoptera frugiperda* (J.E. Smith) (Lepidoptera: Noctuidae), is a highly polyphagous and migratory pest characterized by rapid development, high reproductive capacity, and long-distance dispersal [1,2,3,4]. Its broad host range exposes populations to markedly different nutritional environments and suites of plant defensive chemicals, creating substantial opportunities for host-associated physiological adjustment [5].

Polyphagy does not imply equivalent performance across host plants. Host suitability emerges from the combined effects of feeding acceptance, nutrient composition, physical traits, defensive chemistry, and ecological interactions [6,7,8,9]. Adult preference and offspring performance may also be imperfectly coupled [10]. Consequently, the capacity to feed on a plant is only one component of host use; successful exploitation ultimately depends on whether larvae can develop efficiently, complete metamorphosis, and sustain reproduction. Recent studies of *S. frugiperda* similarly show pronounced host-dependent variation in developmental rate, survival, fecundity, and population growth, emphasizing life-history performance as a direct measure of host adaptation [11,12,13].

Plant specialized metabolites are major determinants of these host-associated differences. Potato, *Solanum tuberosum* L., contains the steroidal glycoalkaloids α-solanine and α-chaconine, two prominent components of its chemical defense that can deter feeding and disrupt membrane-associated and physiological processes in herbivores [14,15,16]. Dietary α-solanine produces dose-dependent effects on insect development, reproduction, and oxidative physiology, while herbivory-induced increases in potato defensive metabolites, including α-solanine and α-chaconine, can suppress subsequent lepidopteran performance [17,18]. The α-solanine component of potato defense therefore provides a tractable chemical axis for examining how sustained exposure reshapes insect performance, while potato foliage places that response within the broader nutritional and defensive environment of the host.

Repeated exposure to plant defensive compounds can favor coordinated changes in xenobiotic handling, including oxidation, hydrolysis, conjugation, transport, and redox homeostasis. Cytochrome P450 monooxygenases are central components of plant–insect chemical interactions, and functional studies in noctuid insects have demonstrated that CYP9A enzymes can enhance protection against xenobiotics [19]. Carboxylesterases, glutathione S-transferases, UDP-glycosyltransferases, and ABC transporters provide complementary biochemical routes for processing, conjugating, and exporting foreign compounds [20]. Recent work in *S. frugiperda* further shows that phytochemical exposure can alter CarE and P450 activities and induce broad transcriptional responses involving detoxification, nutrient metabolism, and stress physiology [21], highlighting the multicomponent nature of adaptation to plant chemistry.

Metabolomics provides a complementary systems-level view of this response because the metabolome integrates genetic background with current physiological and dietary state [22,23,24,25,26,27]. Recent studies of *S. frugiperda* host adaptation have revealed coordinated changes in detoxification-associated genes together with amino-acid, carbohydrate, lipid, and glutathione-related metabolism [28,29]. Feature-level metabolic contrasts and pathway-level inference can therefore reveal whether long-term selection reduces the magnitude of an acute chemical response, redirects metabolic investment, or alters the response to a complete host plant. Integrating these metabolic patterns with life-history traits, enzyme activities, and gene expression provides a direct framework for resolving how insect adaptation to plant defensive chemistry is organized across physiological levels [30,31].

A central question is whether multigenerational selection by a single potato defense compound can generate a coordinated phenotype extending from toxicological tolerance to demographic performance and physiological remodeling. Here, we subjected *S. frugiperda* to 12 generations of selection with α-solanine and compared selected and unselected lines across normal artificial diet, α-solanine-containing diet, and potato foliage. We combined dose–response bioassays, life-history and population analyses, detoxification-enzyme assays, untargeted metabolomics, and targeted RT-qPCR. We predicted that long-term selection would enhance performance under α-solanine exposure, reshape constitutive and diet-responsive detoxification, reduce the magnitude of the acute metabolic response to α-solanine, and modify physiological responses to potato foliage.

## 2. Materials and Methods

### 2.1. Insect Lines, Rearing, and Plant Material

An unselected laboratory line of *Spodoptera frugiperda* was maintained for multiple generations in the College of Plant Protection, Nanjing Agricultural University, and served as the source population for the selection experiment. Larvae were reared at 26 ± 1 °C and approximately 60% relative humidity under a 14 h light:10 h dark photoperiod.

The colony was maintained on a soybean powder– and wheat germ–based artificial diet. One preparation batch contained 450 g soybean powder, 250 g wheat germ, 80 g yeast, 40 g casein, 15 g methylparaben, 0.6 g cholesterol, 6.0 g sorbic acid, 0.7 g myo-inositol, 3.0 g choline chloride, 14 g L(+)-ascorbic acid, 0.15 g penicillin G sodium salt, 60 g agar, 2 mL compound vitamin solution, and 2 mL formaldehyde solution. Soybean powder and wheat germ were mixed with 1200 mL purified water, with additional water added as required during preparation to compensate for evaporation and adjust consistency, and the mixture was sterilized at 120 °C for 20 min. Agar was dissolved separately in 1100 mL of hot purified water. The remaining supplements were dissolved in 500 mL of water at approximately 65 °C and incorporated after the bulk diet had cooled to below 65 °C. The compound vitamin and formaldehyde solutions were then added. The diet was mixed thoroughly, dispensed, allowed to solidify, and stored at 4 °C until use.

Adults were maintained at 70–80% relative humidity and supplied with 10% honey solution. Potato (*Solanum tuberosum* L.) plants were maintained as a laboratory stock and propagated vegetatively for successive generations using tubers produced by the preceding generation. Fresh leaves from this continuously maintained potato stock were used for all potato-foliage feeding assays.

### 2.2. α-Solanine Preparation, Identification, and Content Standardization

The potato-sprout preparation was obtained using a laboratory extraction procedure adapted from the ultrasonic α-solanine extraction method described by Zhou et al. [32]. The preparation was standardized for α-solanine content by targeted LC–ESI–MS/MS. Targeted LC–MS analysis of steroidal glycoalkaloids has previously been applied to Solanum materials [33].

Analyses were performed in positive-ion multiple-reaction-monitoring mode using external α-solanine standards at 0, 50, 100, 200, 300, 400, and 500 ng mL^−1^. The calibration relationship was A = 201.2875C − 261.4220 (R^2^ = 0.9993), where A is peak area, and C is α-solanine concentration. Blank injections produced no integrated target signal under the quantification procedure. The preparation batch used for the bioassays produced the target signal at a retention time of 1.48 min, corresponding to 283.80 ng mL^−1^ in the injected sample. After accounting for the 2500-fold analytical dilution factor, the α-solanine content of the preparation was 0.709 mg mL^−1^. Dietary concentrations are expressed as α-solanine equivalents based on the measured α-solanine content of the standardized preparation. Representative chromatograms and the external calibration are presented in Figure S1.

### 2.3. α-Solanine Selection and Treatment Framework

An α-solanine-selected line was established from the unselected laboratory line and maintained for 12 consecutive generations on an artificial diet containing 1.0 mg α-solanine equivalents g^−1^ diet. This concentration was selected from the initial concentration–response assays to impose a strong, sustained selection pressure while retaining sufficient survivors for continuous colony propagation. Generations 6 and 12 were designated as the intermediate and terminal selection checkpoints, respectively.

Six line-by-diet treatments were established to distinguish line-associated differences from responses to the current diet (Table 1). Treatments A–C comprised the unselected line, whereas treatments D–F comprised the generation-12 selected line. Within each line, insects received a normal artificial diet, an α-solanine-containing artificial diet, or potato foliage.

### 2.4. α-Solanine Bioassay

Dose–response bioassays were conducted at generations 6 and 12. Each well of a 24-well plate received 1.1 mL of artificial diet containing the designated α-solanine concentration. The diet was allowed to solidify before one early second-instar larva was introduced into each well. Two plates were used for each concentration, providing 48 larvae per concentration. After 5 d of exposure, larvae were weighed, and individuals weighing <10 mg were classified as functionally dead, encompassing mortality together with severe feeding and developmental arrest. A comparable growth-based mortality criterion has been used in *S. frugiperda* bioassays [34]. Untreated artificial diet served as the control.

### 2.5. Life-History Measurements

Neonates were maintained individually under the designated line-by-diet treatment. Larvae receiving artificial diets were housed singly in tubes containing freshly prepared diet, whereas larvae receiving potato foliage were maintained individually in Petri dishes with leaf petioles wrapped in moist cotton to preserve turgor. Diet and foliage were renewed as required. After pupation, sex and pupal mass were recorded. Newly emerged females and males were paired and supplied with 10% honey solution; a replacement male was introduced when necessary to maintain mating opportunity. Development and reproduction were recorded twice daily, at 08:00 and 20:00, until death. Recorded endpoints included larval, pupal, adult, and total developmental duration; pupation rate; adult emergence rate; pupal mass; female fecundity; oviposition period; egg hatch; and egg duration.

### 2.6. Traditional Life-Table Calculations

Traditional cohort fertility-table parameters were calculated separately for each biological replicate following the classical demographic framework [35]. At age x, lₓ was the proportion of the initial cohort surviving, mₓ was age-specific egg production per surviving cohort individual, and lₓmₓ represented age-specific reproductive output. Cohort reproductive output was calculated as R_0_ = Σlₓmₓ, mean generation time as T = Σxlₓmₓ/R_0_, intrinsic rate of increase as rₘ = ln(R_0_)/T, finite rate of increase as λ = eʳᵐ, and doubling time as ln(2)/rₘ. Accordingly, R_0_ represents cumulative egg output per initial cohort individual. Treatment means and SEM were calculated from replicate-level parameters. Age-specific survival and fecundity profiles are presented in Figure S2.

### 2.7. Midgut Protein and Detoxification-Enzyme Assays

Fourth-instar larvae from each line-by-diet treatment were exposed to the designated diet for 72 h. Fifteen larvae were sampled per treatment; five midguts were pooled for each biological replicate, providing three biological replicates. Dissected midguts were homogenized in ice-cold 0.1 mol L^−1^ phosphate buffer (pH 7.8), centrifuged at 12,000× *g* for 10 min at 4 °C, and the clarified supernatant was used for protein and enzyme assays.

Protein concentration was determined by a modified Bradford assay using bovine serum albumin as the standard. Carboxylesterase (CarE) activity was measured with 1-naphthyl acetate and calibrated against 1-naphthol; reactions were incubated for 10 min at 30 °C and measured at 600 nm. Activity was expressed as nmol 1-naphthol μg^−1^ protein per 10 min. Cytochrome P450 monooxygenase activity was measured with 4-nitroanisole and NADPH, calibrated against 4-nitrophenol, and measured at 405 nm after 5 min at 30 °C; activity was expressed as nmol 4-nitrophenol μg^−1^ protein per 5 min. Glutathione S-transferase (GST) activity was measured at 340 nm and 30 °C using 1-chloro-2,4-dinitrobenzene and reduced glutathione. Absorbance was recorded every 30 s for 10 min, and activity was calculated using an extinction coefficient of 0.0096 L μmol^−1^ cm^−1^ and expressed as μmol μg^−1^ protein min^−1^.

### 2.8. Untargeted Metabolomics

Early fourth-instar larvae were deprived of food for 4 h and then exposed to the designated diet for 48 h. Three larvae constituted one biological replicate, with six biological replicates per treatment. Samples were extracted with 70% methanol, concentrated, redissolved in methanol, clarified by sequential centrifugation, and filtered through 0.22 μm membranes. A pooled quality-control sample was prepared by combining 20 μL from each biological extract.

Chromatographic separation was performed using an ACQUITY UPLC I-Class PLUS system (Waters, Milford, MA, USA) coupled to a Xevo G2-XS QTOF mass spectrometer (Waters, Milford, MA, USA). An HSS T3 column (2.1 × 100 mm, 1.8 μm) was maintained at 45 °C at a flow rate of 0.4 mL min^−1^. Mobile phase A was water containing 0.1% formic acid, and mobile phase B was acetonitrile containing 0.1% formic acid. The B gradient was 0–2% at 0–1 min, 2–25% at 1–2 min, 25–60% at 2–4 min, 60–90% at 4–7.5 min, 90–99% at 7.5–9.5 min, 99% at 9.5–12.5 min, 99–2% at 12.5–13 min, and 2% at 13–16 min. Electrospray data were acquired in positive- and negative-ion modes over *m*/*z* 50–1200. Source and desolvation temperatures were 120 and 450 °C, respectively; capillary voltage was 3.0 kV in positive-ion mode and 2.0 kV in negative-ion mode. Quality-control performance and the global structure of the metabolomic dataset are shown in Figure S3.

### 2.9. RNA Extraction, cDNA Synthesis, and RT-qPCR

Fourth-instar larvae from each of the six line-by-diet treatments were exposed to the designated diet for 72 h. Fifteen whole larvae were collected per treatment, and five larvae were pooled to form one biological replicate, providing three biological replicates per treatment. Total RNA was extracted and reverse-transcribed into cDNA. RT-qPCR was performed using SYBR chemistry (Vazyme Biotech Co., Ltd., Nanjing, China) on a QuantStudio 7 Pro Real-Time PCR System (Applied Biosystems, Foster, CA, USA), with three technical reactions per biological replicate.

*RPL10* and *RPS24* were used jointly as reference genes. For each sample, the reference Ct was calculated as the mean Ct of *RPL10* and *RPS24*, and relative transcript abundance was determined using the 2^−ΔΔCt^ method [36], with treatment A as the calibrator. The ten-gene panel comprised *SfMaf*; the cytochrome P450 genes *SfCYP9A32*, *SfCYP6AE44*, and *SfCYP6B50*; *SfCPR*; *SfGSTD1*; *SfUGT40L8*; the ABC transporter genes *SfABCC4* and *SfABCG1*; and *SfCAT*. The panel was selected to represent complementary components of xenobiotic and stress physiology, including transcriptional regulation, phase-I oxidation and electron transfer, phase-II conjugation, membrane transport, and oxidative-stress buffering. *RPL10* has shown stable expression under dietary treatments in *S. frugiperda*, whereas *RPS24* ranked among stable reference genes across developmental-stage and temperature comparisons [37]. The use of multiple reference genes reduces dependence on a single reference transcript [38,39]. Primer sequences are provided in Table S1.

### 2.10. Data Analysis

Median lethal concentrations (LC_50_), slopes, and 95% confidence intervals were estimated using PoloPlus (Version 2.0). Tolerance ratios were calculated as the selected-line LC_50_ divided by the contemporaneous unselected-line LC_50_ at the corresponding generation.

Life-history traits, traditional population parameters, and detoxification-enzyme activities were analyzed within the 2 × 3 line-by-diet framework using fixed-effects models containing insect line, diet, and their interaction. Type III sums of squares were used, with biological replicate as the statistical unit. The prespecified same-diet contrasts D−A, E−B, and F−C were adjusted using the Holm–Šidák method within each endpoint.

Raw LC–MS data were processed in Progenesis QI (v3.1) for peak detection, retention-time alignment, and normalization. Principal-component analysis (PCA) and orthogonal partial least-squares discriminant analysis (OPLS-DA) were used for multivariate visualization (Figure S3 and Figure S4). Feature annotations were assigned by comparison with HMDB and METLIN using *m*/*z*, retention time, and available MS/MS information and are reported as putative annotations following metabolomics reporting conventions [30].

For the integrated comparison of line and dietary effects, nine predefined contrasts were evaluated using modeled log_2_ effects, raw *p* values, and Benjamini–Hochberg FDR-adjusted q values. Feature-level responses for the seven primary simple contrasts are summarized in Figure S5 and Figure S6. Representative candidate features shown in Table S2 and Figure S7 were selected from significant, QC-stable features with available putative annotations, after removal of redundant isotope and adduct representations. Features were classified according to the significance and direction of the modeled line × diet interaction effect. Features with FDR q < 0.05 were designated as selected-line-biased when the interaction effect was positive and as unselected-line-biased when it was negative; all remaining features were assigned to the shared/weak-interaction class.

Candidate pathway inference was performed using mummichog 2.7.0 [40]. Positive- and negative-ion datasets were analyzed separately for nine planned contrasts using a custom *Bombyx mori* KEGG azimuth model, a mass tolerance of 10 ppm, a feature cutoff of *p* < 0.05, and 50 permutations. Positive- and negative-ion pathway results were pooled within each contrast and collapsed by pathway name using the smaller *p* value across ion modes. Pathways were assigned to seven predefined physiological contexts. Within each context, the pathway-context support score was calculated as the mean of the three largest −log_10_(P) values, or the mean of all available values when fewer than three pathways were present. In the pathway-context visualization, bubble size represents the number of candidate pathways meeting *p* ≤ 0.05.

RT-qPCR expression values were log_2_-transformed and analyzed separately for each gene using the same two-factor fixed-effects model. For the model-summary matrix, *p* values were adjusted by the Benjamini–Hochberg procedure within each model effect or contrast across the ten genes. The D−A, E−B, and F−C pairwise comparisons were Holm–Šidák adjusted within each gene.

Analyses were performed using PoloPlus (Version 2.0), SPSS 25.0, mummichog 2.7.0, and Python 3.11.15 with SciPy and statsmodels. Unless otherwise stated, statistical significance was accepted at *p* < 0.05.

## 3. Results

### 3.1. Long-Term α-Solanine Selection Increased Tolerance and Altered Life-History Traits

**Figure 1:**
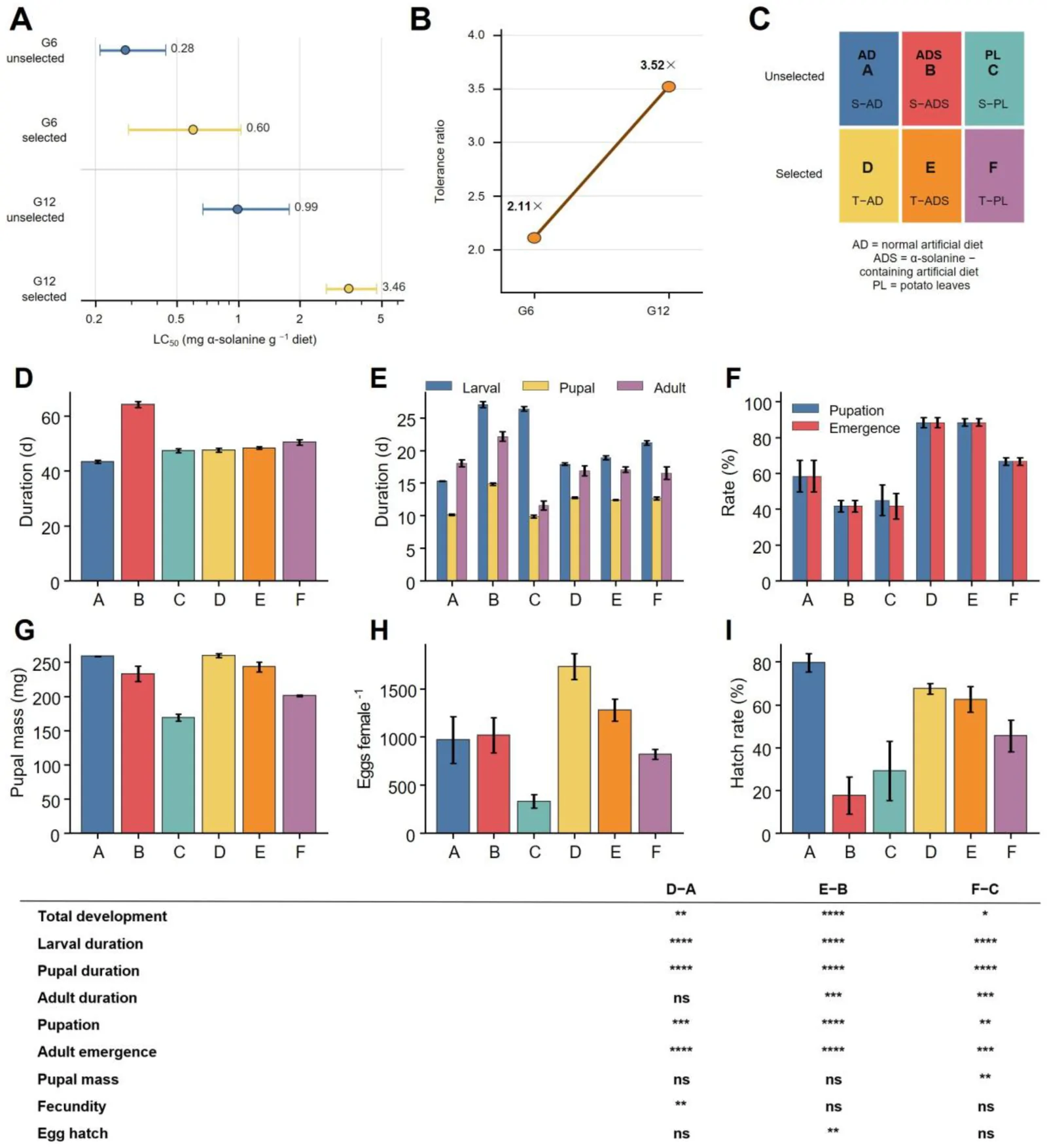
Development of α-solanine tolerance and life-history responses in unselected and selected *Spodoptera frugiperda* lines. (**A**) LC_50_ estimates and 95% confidence intervals for the unselected and selected lines at generations 6 and 12. (**B**) Tolerance ratios of the selected line relative to the contemporaneous unselected line. (**C**) Experimental framework defining the six line-by-diet treatments. (**D**) Total developmental duration. (**E**) Larval, pupal, and adult duration. (**F**) Pupation and adult emergence rates. (**G**) Pupal mass. (**H**) Female fecundity. (**I**) Egg hatch rate. In panels (**D**–**I**), bars show biological-replicate means ± SEM. The matrix below the panels summarizes the prespecified same-diet comparisons D−A, E−B, and F−C from the two-factor model, with Holm–Šidák adjustment within each endpoint. S, unselected laboratory line; T, α-solanine-selected line; AD, normal artificial diet; ADS, α-solanine-containing artificial diet; PL, potato foliage. ns, not significant; * *p* < 0.05; ** *p* < 0.01; *** *p* < 0.001; **** *p* < 0.0001.

The selected line showed higher α-solanine tolerance at both assay generations (Figure 1A,B). At generation 6, the LC_50_ was 0.28 mg g^−1^ diet (95% CI, 0.21–0.44) in the unselected line and 0.60 mg g^−1^ diet (0.29–1.03) in the selected line, corresponding to a tolerance ratio of 2.11. At generation 12, LC_50_ values were 0.99 mg g^−1^ diet (0.67–1.77) and 3.46 mg g^−1^ diet (2.69–4.75), respectively, with a tolerance ratio of 3.52.

Life-history traits differed most strongly between lines under the α-solanine-containing diet (Figure 1C–I). Total developmental duration was 64.13 d in the unselected line (B) and 48.35 ± 0.51 d in the selected line (E). Larval, pupal, adult, and total developmental durations were all significantly shorter in E than in B. Pupation increased from 43.33% in B to 88.33 ± 2.11% in E, and adult emergence increased from 41.67% to 88.33 ± 2.11%.

Pupal mass did not differ significantly between E and B (E: 242.96 ± 6.86 mg; Holm–Šidák-adjusted *p* = 0.398). Fecundity was 1280.88 ± 114.26 eggs female^−1^ in E and did not differ significantly from B (adjusted *p* = 0.230). Egg hatch increased from 17.7% in B to 62.56 ± 5.99% in E (adjusted *p* = 0.0021). The oviposition period and egg duration in E were 4.58 ± 0.08 d and 3.95 ± 0.21 d, respectively.

### 3.2. Population Parameters Differed Between Selected and Unselected Lines

Population parameters were calculated for all six line-by-diet treatments (Table S3; Figure S2). Under the α-solanine-containing diet, mean generation time decreased from 52.67 ± 2.04 d in B to 35.88 ± 0.38 d in E. The intrinsic rate of increase increased from 0.1019 ± 0.0063 d^−1^ to 0.1484 ± 0.0066 d^−1^, and the finite rate of increase increased from 1.1073 ± 0.0069 d^−1^ to 1.1601 ± 0.0076 d^−1^. Doubling time decreased from 6.86 ± 0.45 d to 4.72 ± 0.21 d. These four E−B contrasts were significant. R_0_ was 216.68 ± 41.20 cumulative eggs per initial cohort individual in B and 228.93 ± 46.11 in E and did not differ significantly between lines.

### 3.3. Detoxification-Enzyme Activities Varied with Selection History and Diet

**Figure 2:**
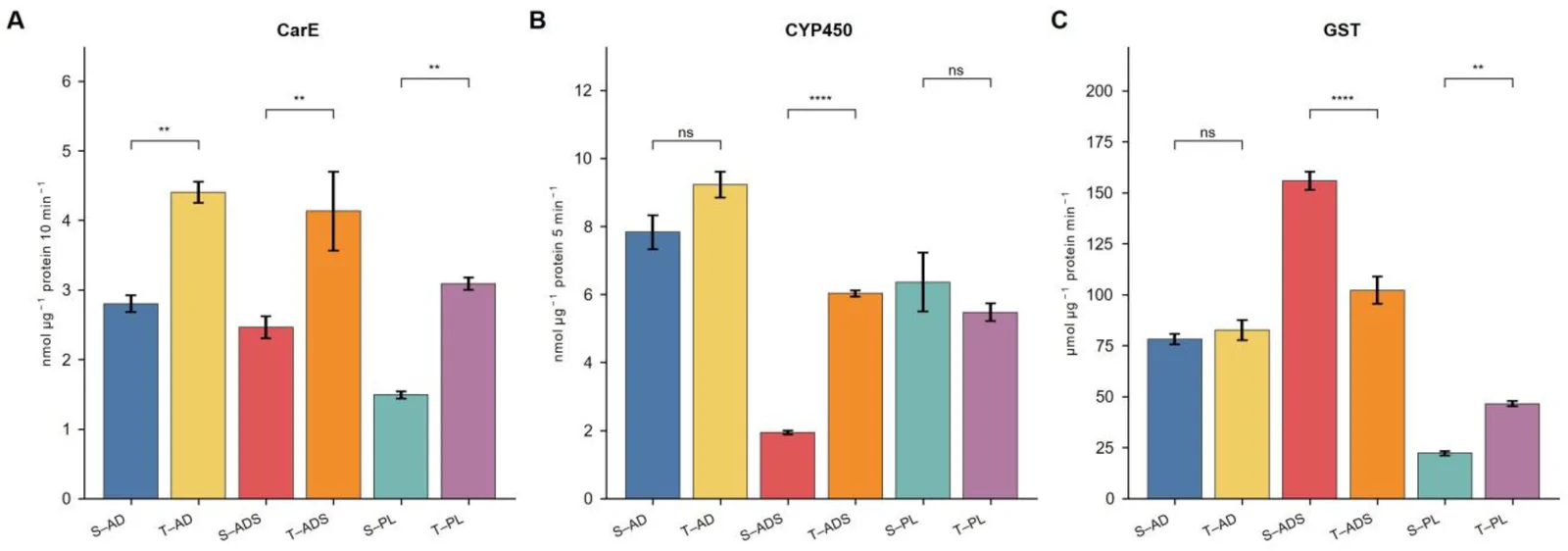
Detoxification-enzyme activities of unselected and α-solanine-selected lines under three dietary conditions. (**A**) Carboxylesterase (CarE), (**B**) cytochrome P450 monooxygenase (CYP450), and (**C**) glutathione S-transferase (GST) activities. Bars show means ± SEM for three biological replicates. Brackets indicate Holm–Šidák-adjusted planned comparisons between the unselected and selected lines within the same diet. S, unselected laboratory line; T, α-solanine-selected line; AD, normal artificial diet; ADS, α-solanine-containing artificial diet; PL, potato foliage. ns, not significant; ** *p* < 0.01; **** *p* < 0.0001.

CarE activity showed significant effects of insect line (F_1_,_12_ = 59.59, *p* = 5.41 × 10^−6^) and diet (F_2_,_12_ = 14.17, *p* = 0.0007), whereas the line × diet interaction was nonsignificant (F_2_,_12_ = 0.01, *p* = 0.988; Figure 2A). CarE activity was significantly higher in the selected line under AD, ADS, and PL.

CYP450 activity was affected by line (F_1_,_12_ = 17.44, *p* = 0.0013), diet (F_2_,_12_ = 51.46, *p* = 1.30 × 10^−6^), and their interaction (F_2_,_12_ = 15.36, *p* = 0.0005; Figure 2B). CYP450 activity differed significantly between E and B, whereas D−A and F−C were nonsignificant.

GST activity showed significant line (F_1_,_12_ = 6.30, *p* = 0.0274), diet (F_2_,_12_ = 270.65, *p* = 1.04 × 10^−10^), and line × diet effects (F_2_,_12_ = 49.64, *p* = 1.57 × 10^−6^; Figure 2C). GST activity differed significantly between E and B and between F and C, but not between D and A.

### 3.4. The Metabolomic Response to α-Solanine Was Reduced in the Selected Line

**Figure 3:**
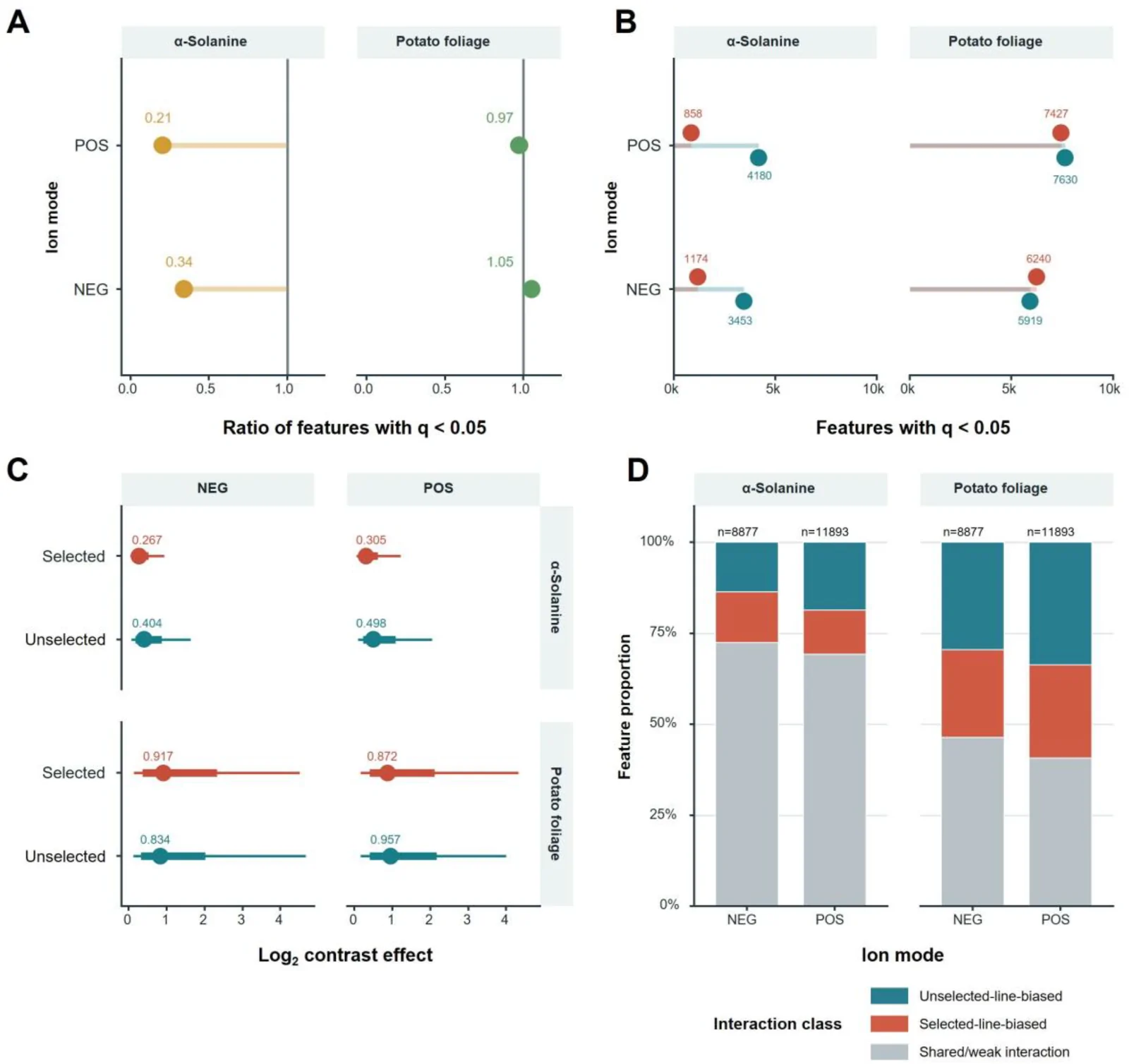
Magnitude and structure of untargeted metabolomic responses to α-solanine exposure and potato foliage. (**A**) Ratios of responsive features (q < 0.05) in the selected line relative to the unselected line under α-solanine exposure and potato foliage, shown separately for positive- and negative-ion modes. (**B**) Numbers of responsive features in the selected and unselected lines under the two dietary challenges. (**C**) Distributions of the absolute modeled log_2_ contrast effects for each line under α-solanine exposure and potato foliage; numerical labels indicate median absolute effects. (**D**) Proportional composition of unselected-line-biased, selected-line-biased, and shared/weak interaction classes among detected features. POS and NEG denote positive- and negative-ion modes, respectively. S, unselected laboratory line; T, α-solanine-selected line; AD, normal artificial diet; ADS, α-solanine-containing artificial diet; PL, potato foliage.

The number of α-solanine-responsive features was lower in the selected line than in the unselected line in both ion modes (Figure 3A,B). The selected-to-unselected ratios of features with FDR q < 0.05 were 0.21 in positive-ion mode and 0.34 in negative-ion mode. Under α-solanine exposure, 858 positive-ion and 1174 negative-ion features were responsive in the selected line, compared with 4180 and 3453, respectively, in the unselected line.

The corresponding ratios under potato foliage were 0.97 in positive-ion mode and 1.05 in negative-ion mode (Figure 3A). Responsive-feature counts under potato foliage were 7427 and 6240 in the selected line and 7630 and 5919 in the unselected line for positive- and negative-ion modes, respectively (Figure 3B).

Feature-level effect distributions also differed between α-solanine and potato-foliage responses (Figure 3C). Under α-solanine exposure, median absolute log_2_ effects were 0.305 in positive-ion mode and 0.267 in negative-ion mode in the selected line, compared with 0.498 and 0.404 in the unselected line. Under potato foliage, the corresponding medians were 0.872 and 0.917 in the selected line and 0.957 and 0.834 in the unselected line.

Representative significant features across the seven primary contrasts are shown in Table S2 and Figure S7. Putatively annotated features included solanidine, Gamma2-Solamarine, phospholipid-related features, and pheophorbide-related features, with both positive and negative modeled effects across contrasts.

### 3.5. Pathway-Context Profiles Differed Across Line and Diet Contrasts

**Figure 4:**
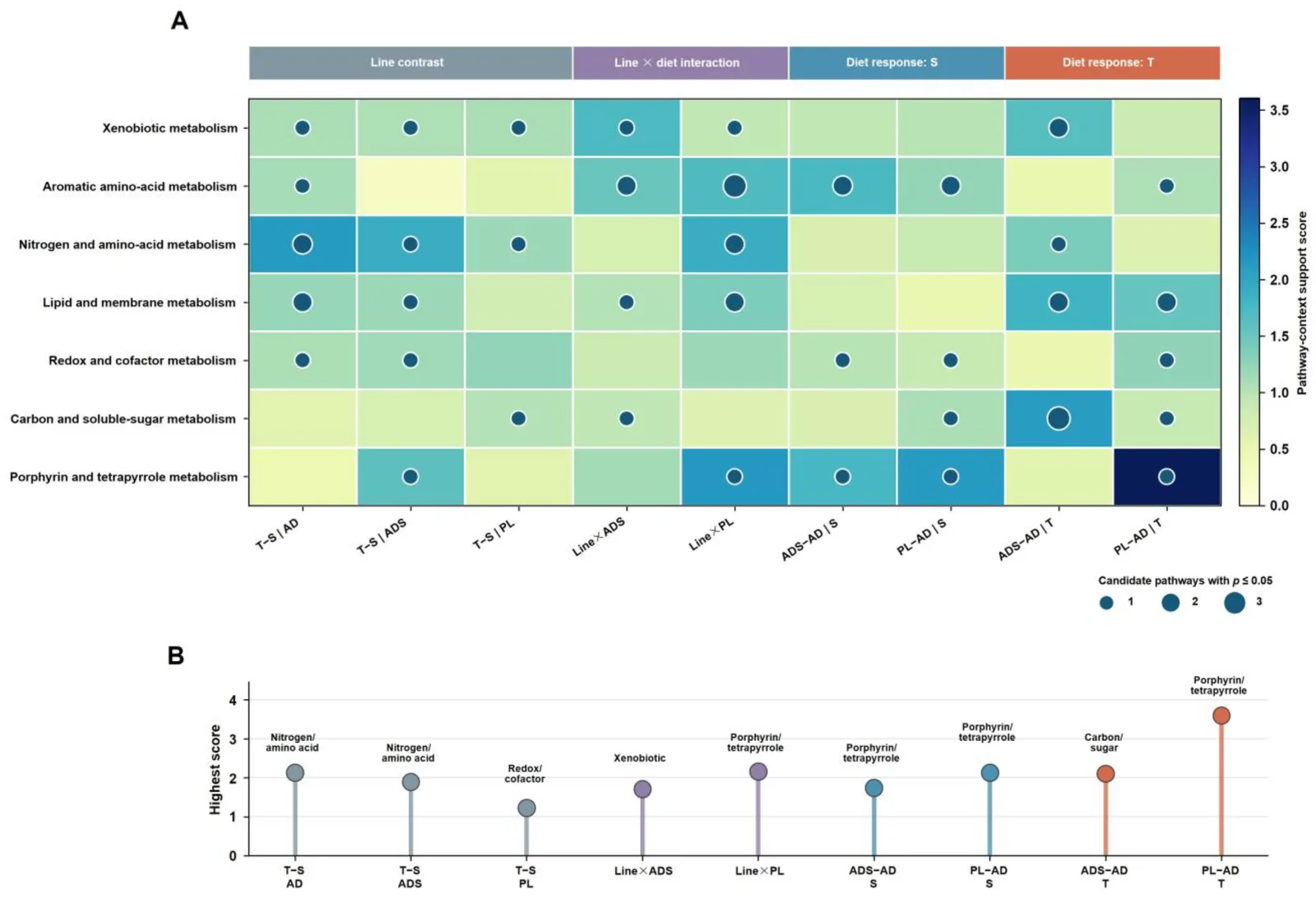
Candidate pathway-context profiles across line and diet contrasts. (**A**) Pathway-context support matrix for the nine predefined contrasts. Tile color represents the pathway-context support score, whereas bubble size represents the number of candidate pathways meeting *p* ≤ 0.05; cells without a bubble had no candidate pathway meeting this threshold. (**B**) Highest-scoring physiological context for each contrast, with lollipop height representing the corresponding pathway-context score. S, unselected laboratory line; T, α-solanine-selected line; AD, normal artificial diet; ADS, α-solanine-containing artificial diet; PL, potato foliage.

The nine predefined contrasts produced distinct pathway-context profiles (Figure 4A). For the three selected-versus-unselected comparisons, nitrogen and amino-acid metabolism was the highest-scoring context under AD and ADS, whereas redox and cofactor metabolism ranked highest under PL (Figure 4B). Xenobiotic metabolism was the highest-scoring context for the line × ADS interaction, while porphyrin and tetrapyrrole metabolism ranked highest for the line × PL interaction.

Within the unselected line, porphyrin and tetrapyrrole metabolism was the highest-scoring context for both ADS−AD and PL−AD. Within the selected line, carbon and soluble-sugar metabolism ranked highest for ADS−AD, whereas porphyrin and tetrapyrrole metabolism ranked highest for PL−AD.

### 3.6. Selection Altered Constitutive and Diet-Responsive Gene Expression

**Figure 5:**
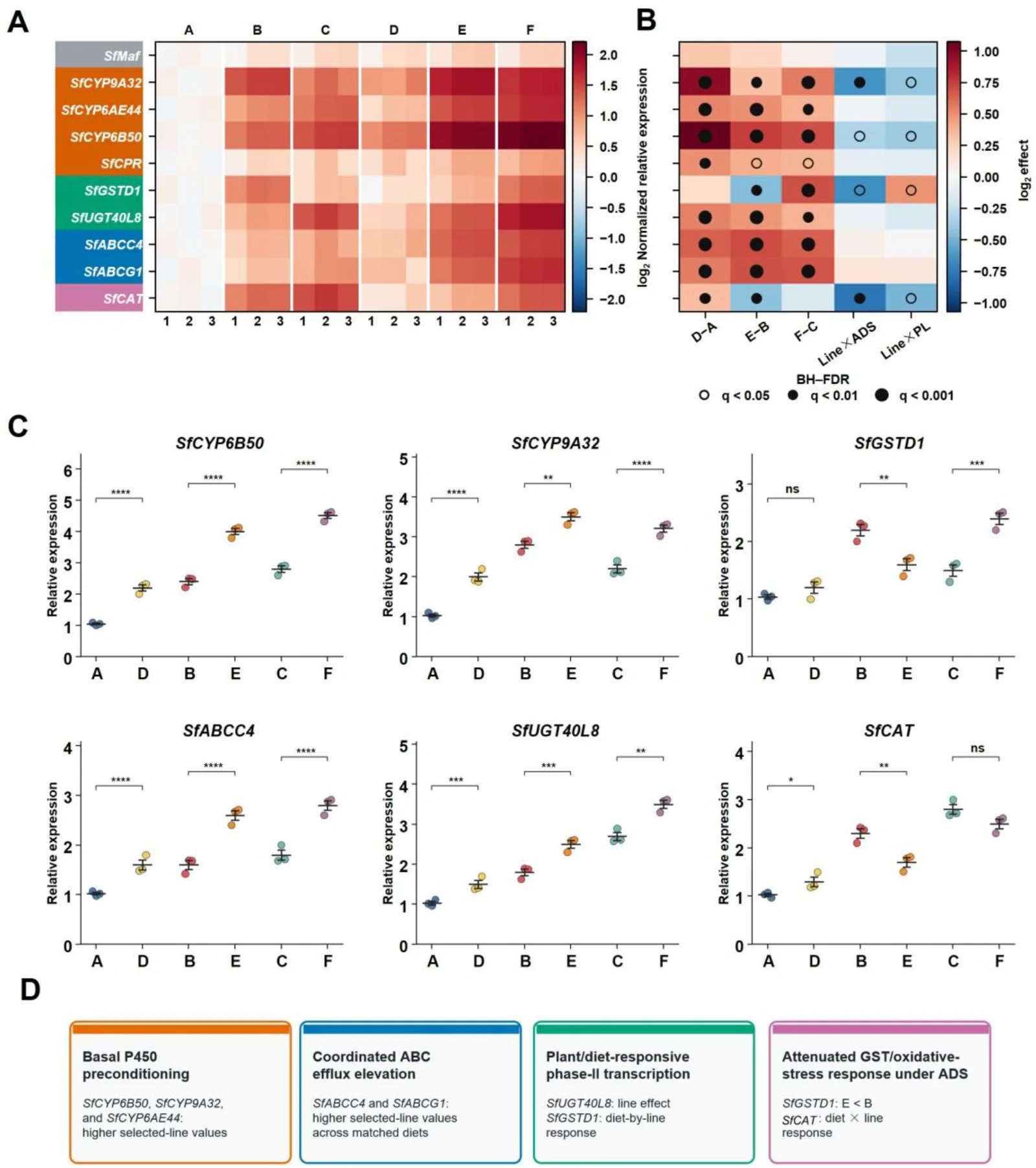
Constitutive and diet-dependent expression patterns of detoxification- and stress-associated genes. (**A**) Heatmap of log_2_-normalized relative expression for ten candidate genes across three biological replicates of each of the six treatments. (**B**) Modeled log_2_ effects for the three same-diet line contrasts (D−A, E−B, and F−C) and the line × diet interaction terms for α-solanine diet and potato foliage. Circle symbols indicate Benjamini–Hochberg FDR significance: open circles, q < 0.05; filled circles, q < 0.01; larger filled circles, q < 0.001. (**C**) Relative expression of six representative genes (*SfCYP6B50*, *SfCYP9A32*, *SfGSTD1*, *SfABCC4*, *SfUGT40L8*, and *SfCAT*) shown in paired treatment order A–D–B–E–C–F. Points represent biological replicates, horizontal bars show means ± SEM, and brackets indicate Holm–Šidák-adjusted same-diet comparisons. (**D**) Graphical summary of the principal constitutive and diet-responsive expression patterns across the candidate-gene panel. Relative expression was normalized jointly to *RPL10* and *RPS24*, with treatment A as the calibrator. S, unselected laboratory line; T, α-solanine-selected line; AD, normal artificial diet; ADS, α-solanine-containing artificial diet; PL, potato foliage. ns, not significant; * *p* < 0.05; ** *p* < 0.01; *** *p* < 0.001; **** *p* < 0.0001.

RT-qPCR revealed significant line, diet, and interaction effects across multiple detoxification- and stress-associated genes (Figure 5A,B). Among the six representative genes shown in Figure 5C, *SfCYP6B50*, *SfCYP9A32*, *SfABCC4*, and *SfUGT40L8* were expressed at significantly higher levels in the selected line under all three matched diets.

*SfGSTD1* did not differ between D and A, was significantly lower in E than in B, and was significantly higher in F than in C. The E/B expression ratio was 0.73, whereas the F/C ratio was 1.60.

*SfCAT* expression was significantly higher in D than in A, significantly lower in E than in B, and did not differ significantly between F and C. *SfMaf* showed no FDR-supported model effect (Figure 5B).

## 4. Discussion

Twelve generations of α-solanine selection produced a tolerance ratio of 3.52 and a coordinated phenotype spanning development, demographic performance, detoxification enzymes, metabolomic responses, and gene expression. The clearest divergence between selected and unselected lines occurred under the α-solanine-containing diet, where selection shortened development and substantially increased pupation, adult emergence, and egg hatch. These changes were accompanied by shorter generation time, higher *r*ₘ and λ, and a shorter doubling time, indicating that repeated exposure to α-solanine reshaped both toxicological tolerance and population performance.

The life-history response is consistent with growing evidence that plant chemistry can strongly influence *S. frugiperda* development and demography [41]. Dietary α-solanine causes dose-dependent developmental, reproductive, and oxidative responses in lepidopteran larvae [17], while induced changes in potato defensive chemistry can suppress subsequent herbivore growth [18]. In *S. frugiperda*, host plants differing in nutritional and chemical composition produce pronounced variation in developmental rate, pupation, fecundity, and population-growth parameters [11,12,13]. Recent comparisons among maize, soybean, and sweet potato similarly showed large host-associated differences in *R*_0_, *r*ₘ, λ, and generation time [12]. The present selection experiment adds an evolutionary dimension to these host effects: under the α-solanine diet, the selected line not only survived the challenge more effectively but completed development faster and maintained higher demographic growth indices.

The contrast between the α-solanine-containing diet and intact potato foliage further illustrates how adaptation to one defensive compound becomes embedded within the broader host environment. Potato foliage contains α-solanine together with α-chaconine and other specialized metabolites, as well as a distinct nutritional and structural matrix. α-Solanine and α-chaconine are closely related steroidal glycoalkaloids, and both can be degraded through overlapping deglycosylation reactions in a potato-feeding lepidopteran-associated gut bacterium [42]. In the present study, selection generated clear gains under direct α-solanine exposure and also altered performance and physiological responses on potato foliage, indicating that adaptation to a major component of host defense can influence the response to the more complex chemical environment of the whole plant.

The enzyme and transcriptional data reveal both stable and diet-responsive components of this selected phenotype. CarE activity showed a strong overall line effect and remained higher in the selected line across all three diets, whereas CYP450 and GST activities were strongly shaped by current diet and line × diet interactions. A similar combination of constitutive and inducible detoxification has been reported in *S. frugiperda* exposed to plant-derived toxicants. Plumbagin treatment, for example, increased CarE and P450 activities while inducing broad transcriptional changes involving xenobiotic detoxification, nutrient metabolism, and stress physiology [21]. Comparative genomic analyses have also revealed substantial variation among *Spodoptera* lineages in CYP, COE, GST, UGT, and ABC gene families associated with host use [43]. Together with functional evidence for CYP9A-mediated xenobiotic protection [19] and validated detoxification genes in *S. frugiperda* [20], these findings place the enzyme responses observed here within a broader architecture of constitutive capacity and diet-dependent regulation.

The transcriptional profile extends this pattern beyond phase-I metabolism. *SfUGT40L8*, *SfABCC4*, and *SfABCG1* showed elevated expression in the selected line across matched diets, consistent with coordinated changes in conjugation and transport-related capacity. The elevated expression of *SfUGT40L8* is particularly notable in light of functional studies showing that members of the expanded UGT40 family contribute to xenobiotic detoxification in *S. frugiperda* [44]. In parallel, the UGT33 paralogs SfruUGT33T10 and SfruUGT33F32 directly glycosylate maize benzoxazinoids and contribute to host-plant tolerance [45]. These findings place the present *SfUGT40L8* response within a broader functional role of expanded UGT families in chemical adaptation. The present expression patterns therefore fit a broader model in which long-term exposure to plant defensive chemistry recruits multiple phases of xenobiotic handling rather than relying on a single detoxification route. The contrasting responses of *SfGSTD1* and *SfCAT* across α-solanine diet and potato foliage further emphasize that this selected state retains strong sensitivity to current dietary context.

The metabolomic response showed a parallel shift. Upon renewed α-solanine exposure, the selected line exhibited substantially fewer responsive features and smaller median effect magnitudes than the unselected line, whereas responses to potato foliage remained broader and more comparable between lines. This reduced feature-level displacement is consistent with a physiological state that has become less perturbed by the selection compound. Recent multi-omics studies of *S. frugiperda* host adaptation similarly link host-associated performance with coordinated changes in detoxification genes, enzyme activities, and amino-acid, carbohydrate, lipid, glutathione, and related metabolic pathways [28,29]. The convergence between these studies and the present results lies in the coordinated remodeling of metabolic and detoxification processes during adaptation to chemically distinct diets.

Pathway-context analysis reinforced this systems-level organization. Line contrasts, line × diet interactions, and acute dietary responses were associated with different dominant physiological contexts, including xenobiotic metabolism, amino-acid and nitrogen metabolism, redox and cofactor metabolism, porphyrin and tetrapyrrole metabolism, and carbon and soluble-sugar metabolism. Combined with the reduced feature burden under α-solanine exposure, the enzyme profiles, and the transcriptional shifts in P450, UGT, ABC, GST, and oxidative-stress genes, these patterns show that selection reorganized the physiological response across multiple metabolic layers.

More broadly, these findings demonstrate that sustained selection by a single plant defensive compound can generate phenotypic changes extending well beyond a conventional dose–response shift. In *S. frugiperda*, α-solanine selection altered developmental timing, metamorphic success, demographic performance, detoxification physiology, metabolic responsiveness, and transcriptional state. The persistence of distinct responses on potato foliage further shows that adaptation to one defensive compound can modify, rather than simply replace, the physiological response to a complex host environment. Such multilevel plasticity and selection responses may contribute to the capacity of highly polyphagous insects to exploit chemically heterogeneous crop landscapes.

## 5. Conclusions

Twelve generations of α-solanine selection increased tolerance in *Spodoptera frugiperda* and produced coordinated changes in life-history performance, demographic growth, detoxification physiology, metabolism, and gene expression. Under α-solanine exposure, the selected line developed more rapidly, achieved higher pupation and adult emergence, showed increased egg hatch and faster demographic turnover, and exhibited a markedly reduced metabolomic response. CarE activity showed a strong line-associated increase, whereas CYP450, GST, and several detoxification- and stress-related genes retained pronounced diet-dependent responses. Together, these changes define a multilevel adaptive phenotype in which long-term selection by a single plant defensive compound reshaped both α-solanine tolerance and the physiological response to potato foliage.

## Figures and Tables

**Table 1:** Definitions of the six line-by-diet treatments used throughout the study.

Treatment	Insect Line	Diet Treatment
A	Unselected (S)	Normal artificial diet (AD)
B	Unselected (S)	α-Solanine-containing artificial diet (ADS; 1.0 mg α-solanine equivalents g^−1^ diet)
C	Unselected (S)	Potato foliage (PL)
D	Selected (T; G12)	Normal artificial diet (AD)
E	Selected (T; G12)	α-Solanine-containing artificial diet (ADS; 1.0 mg α-solanine equivalents g^−1^ diet)
F	Selected (T; G12)	Potato foliage (PL)

## Data Availability

Data generated in association with this study are available in the Supplementary Materials online with this article.

## Supplementary Materials

The following supporting information can be downloaded at: https://www.mdpi.com/article/10.3390/insects17090941/s1. Figure S1: Targeted LC–ESI–MS/MS characterization and α-solanine content standardization of the potato-sprout-derived preparation used for the bioassays. Figure S2: Age-specific survival and fecundity profiles of unselected and selected lines across the three dietary conditions. Figure S3: Quality control and global structure of the untargeted metabolomic dataset. Figure S4: OPLS-DA score plots for line and dietary contrasts in positive- and negative-ion modes. Figure S5: Feature-level responses across seven primary metabolomic contrasts. Figure S6: Responsive-feature counts, intersections, and abundance patterns across the six treatments. Figure S7: Representative differentially abundant candidate metabolic features across the seven primary contrasts. Table S1: Primer sequences used for RT-qPCR analysis. Table S2: Representative differentially abundant candidate metabolic features across the seven primary contrasts. Table S3: Traditional cohort fertility-table parameters for treatments A–F.
