# Supplementary material for: Long-Term Selection with α-Solanine Enhances Tolerance and Drives Multilevel Physiological Remodeling in Spodoptera frugiperda (J.E. Smith) (Lepidoptera: Noctuidae)
Source: Insects. 2026 Sep 8;17(9):941. doi: 10.3390/insects17090941 (PMC13607215; doi:10.3390/insects17090941)
Supplement: Supplementary file 1 [file insects-17-00941-s001.zip › insects-4524853-supplementary.pdf]

Supplementary data

Long-Term Selection with α-Solanine Enhances Tolerance and Drives Multilevel Physiological Remodeling in *Spodoptera frugiperda* (J.E. Smith) (Lepidoptera: Noctuidae)

Yi-Kuan Wu [1], Peng-Fei Zhang [1], Jia-Qi Wu [1], Bo Pang [2], Guo-Qing Li [1] and Lin Jin [1,*]

[1] State Key Laboratory of Agricultural and Forestry Biosecurity, College of Plant Protection, Nanjing Agricultural University, Nanjing 210095, China; 2023202051@stu.njau.edu.cn (Y.-K.W.); 2020102079@stu.njau.edu.cn (P.-F.Z.); 2026002008@stu.njau.edu.cn (J.-Q.W.); ligq@njau.edu.cn (G.-Q.L.)

[2] Institute of Agriculture, Xizang Academy of Agricultural and Animal Husbandry Sciences, Lasa 850000, China; 13989010631@163.com

* Correspondence: jinlin@njau.edu.cn

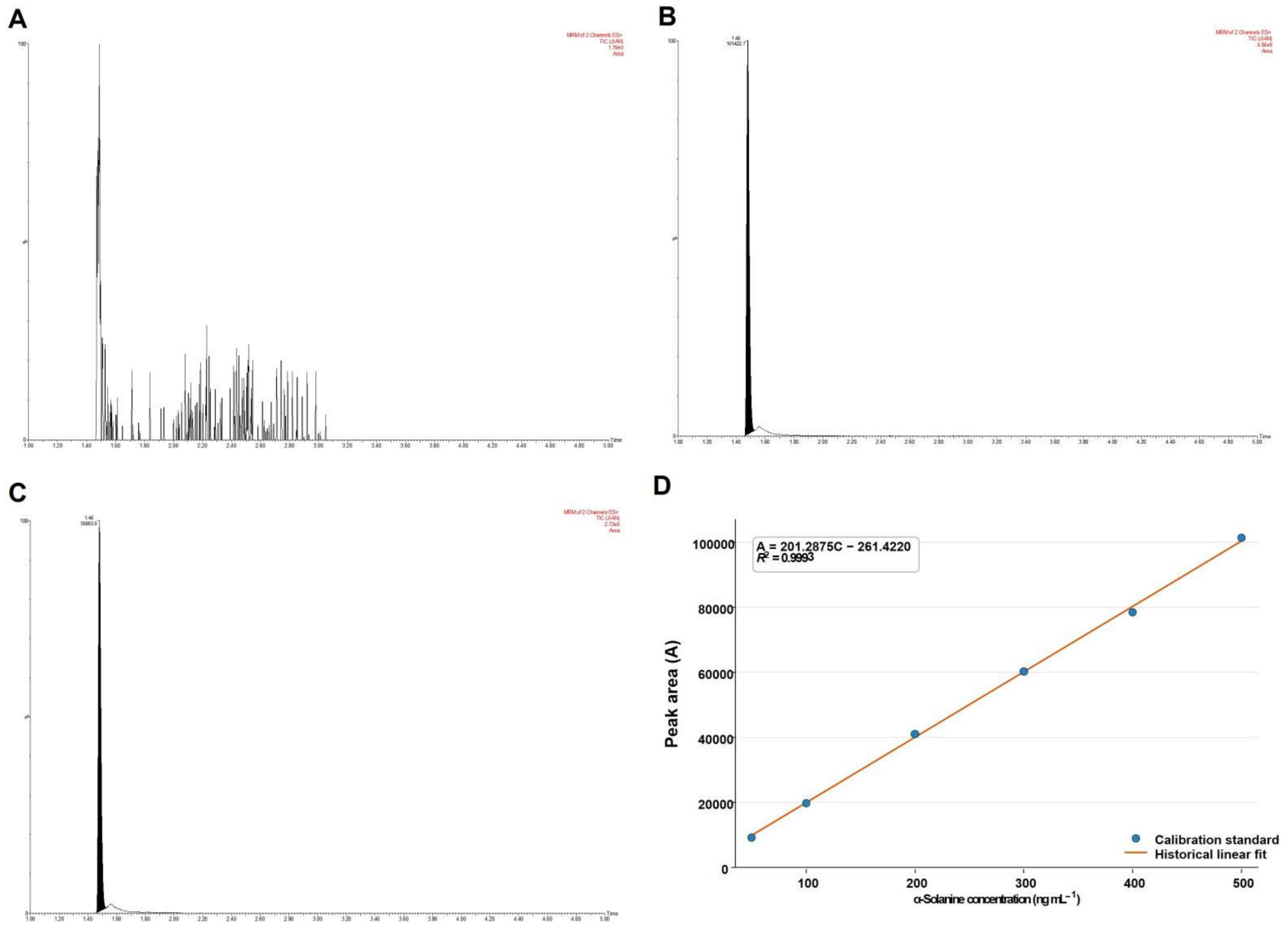

Figure S1. Targeted LC–ESI–MS/MS characterization and α-solanine content standardization of the potato-sprout-derived preparation used for the bioassays. (A) Blank injection chromatogram; no target peak was integrated by the quantification procedure. (B) α-Solanine standard at 500 ng mL^{-1}, showing the target signal at approximately 1.48 min. (C) Formal α-solanine-standardized preparation used for the bioassays, showing the corresponding target signal at approximately 1.48 min. (D) External α-solanine calibration over 50–500 ng mL^{-1}; the fitted relationship was A = 201.2875C − 261.4220 (R^2 = 0.9993), where A is peak area and C is α-solanine concentration.

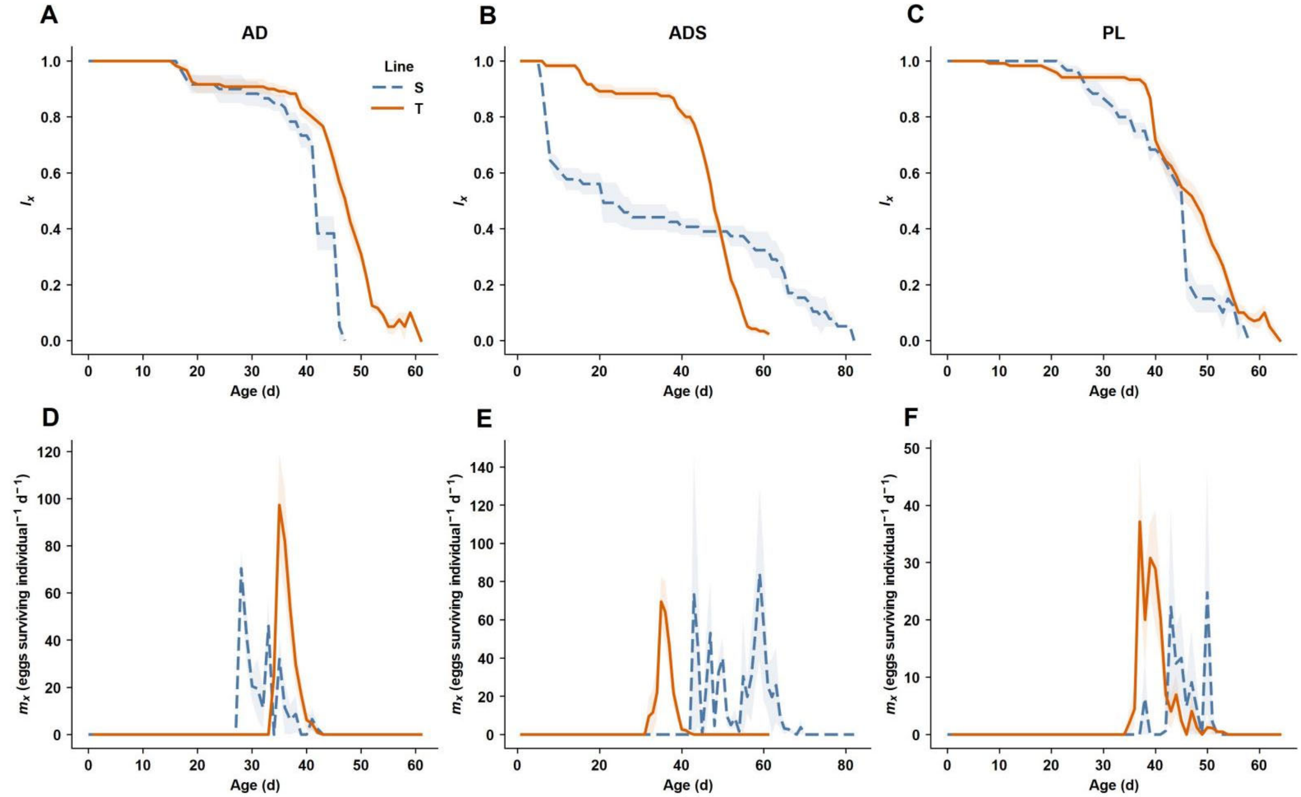

Figure S2. Age-specific survival and fecundity profiles of unselected and selected lines across the three dietary conditions. (A–C) Age-specific survival (l_x) under normal artificial diet (AD), α-solanine-containing artificial diet (ADS), and potato foliage (PL), respectively. (D–F) Age-specific fecundity (m_x) under AD, ADS, and PL, respectively. Dashed and solid lines represent the unselected (S) and selected (T) lines, respectively; shaded bands indicate SEM among biological replicates. m_x denotes age-specific egg production per surviving cohort individual. S, unselected laboratory line; T, α-solanine-selected line.

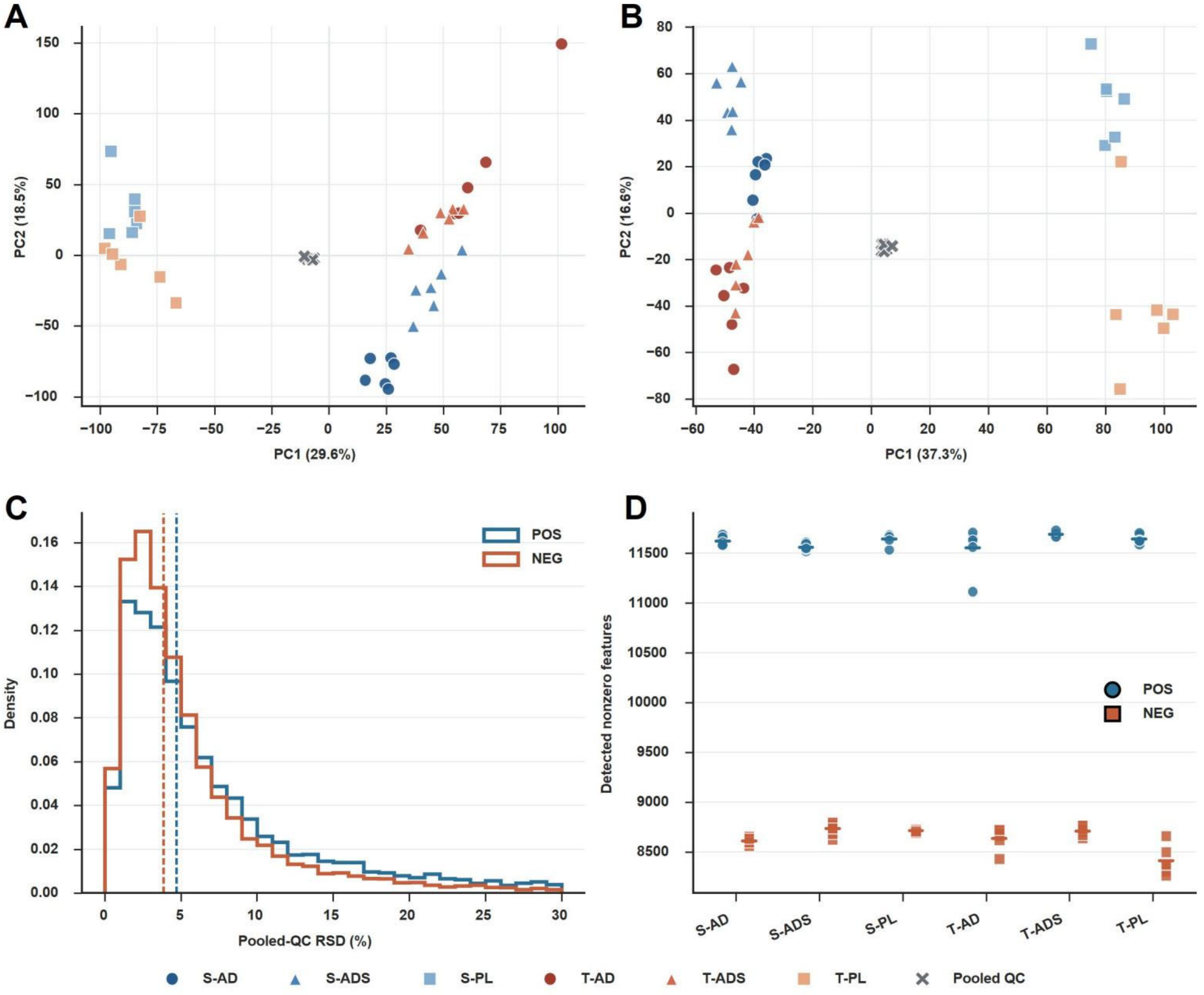

Figure S3. Quality control and global structure of the untargeted metabolomic dataset. (A,B) Principal-component analysis of biological samples and pooled quality-control (QC) samples in positive- and negative-ion modes, respectively. Colors distinguish the unselected and selected lines, symbol shapes distinguish diets, and gray crosses denote pooled-QC samples; percentages on the axes indicate the variance explained by each principal component. (C) Distribution of relative standard deviations (RSDs) among pooled-QC features in positive- and negative-ion modes; dashed vertical lines indicate the corresponding median RSDs. (D) Numbers of non-zero features detected across biological replicates of the six treatments. S, unselected laboratory line; T, α-solanine-selected line; AD, normal artificial diet; ADS, α-solanine-containing artificial diet; PL, potato foliage; POS and NEG, positive- and negative-ion modes, respectively.

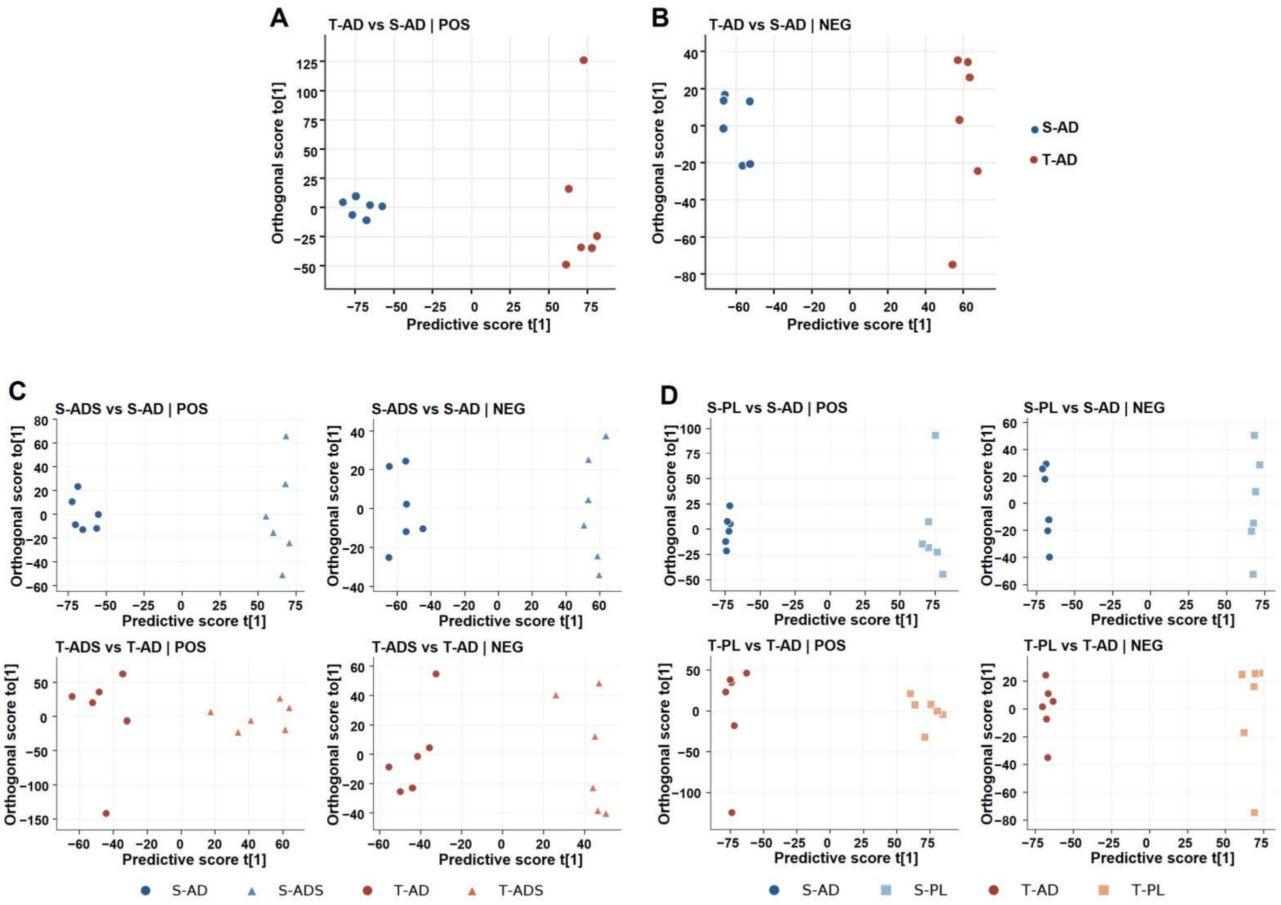

Figure S4. OPLS-DA score plots for line and dietary contrasts in positive- and negative-ion modes. (A,B) Selected versus unselected lines under the normal artificial diet (T-AD vs. S-AD) in positive- and negative-ion modes, respectively. (C) α-Solanine-diet versus normal-diet contrasts within the unselected line (S-ADS vs. S-AD) and selected line (T-ADS vs. T-AD), shown separately for positive- and negative-ion modes. (D) Potato-foliage versus normal-diet contrasts within the unselected line (S-PL vs. S-AD) and selected line (T-PL vs. T-AD), shown separately for positive- and negative-ion modes. Each point represents one biological replicate. The horizontal and vertical axes represent the predictive component t[1] and orthogonal component to[1], respectively. S, unselected laboratory line; T, α-solanine-selected line; AD, normal artificial diet; ADS, α-solanine-containing artificial diet; PL, potato foliage.

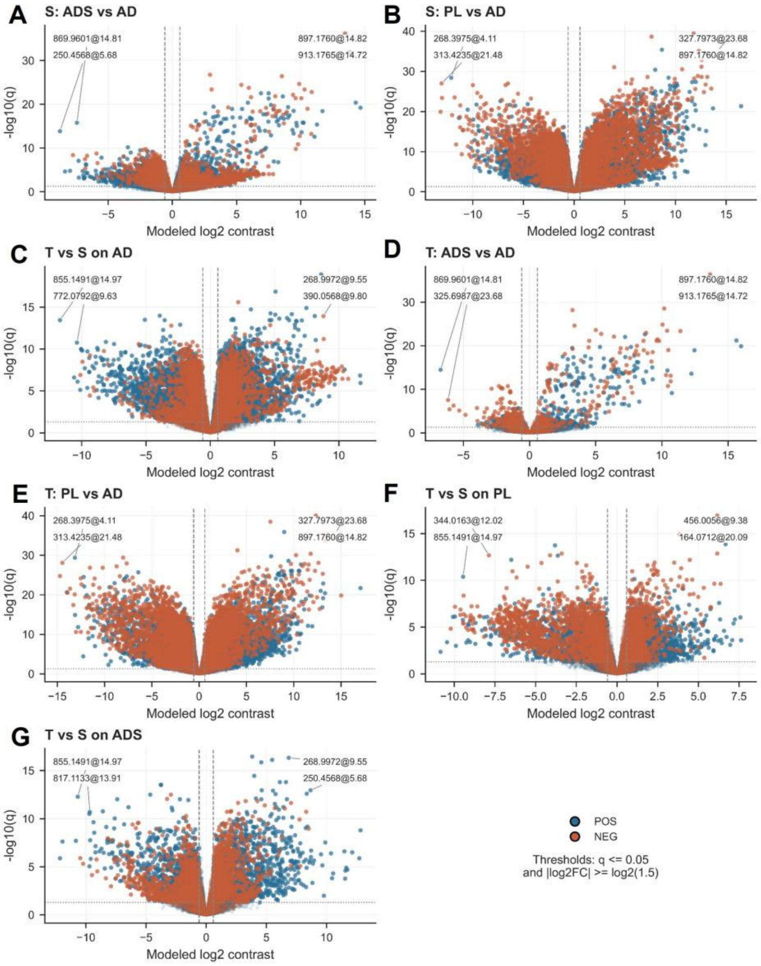

Figure S5. Feature-level responses across seven primary metabolomic contrasts. (A) α-Solanine-containing versus normal artificial diet in the unselected line (S-ADS vs. S-AD). (B) Potato foliage versus normal artificial diet in the unselected line (S-PL vs. S-AD). (C) Selected versus unselected lines under normal artificial diet (T-AD vs. S-AD). (D) α-Solanine-containing versus normal artificial diet in the selected line (T-ADS vs. T-AD). (E) Potato foliage versus normal artificial diet in the selected line (T-PL vs. T-AD). (F) Selected versus unselected lines under potato foliage (T-PL vs. S-PL). (G) Selected versus unselected lines under the α-solanine-containing diet (T-ADS vs. S-ADS). Positive- and negative-ion features are shown in blue and orange, respectively. Dashed vertical lines indicate $|\log_2 FC| = \log_2(1.5)$, and the horizontal dotted line indicates FDR q = 0.05. Labels identify selected features by m/z@retention time. S, unselected laboratory line; T, α-solanine-selected line; AD, normal artificial diet; ADS, α-solanine-containing artificial diet; PL, potato foliage.

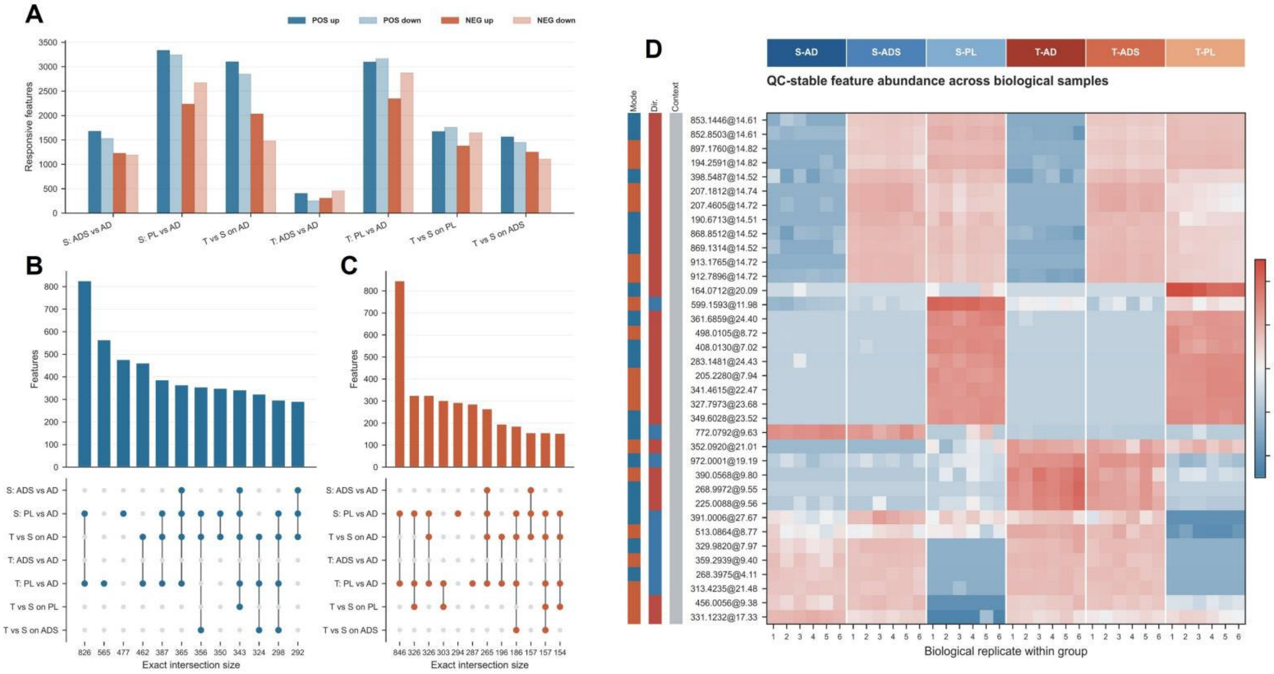

Figure S6. Responsive-feature counts, intersections, and abundance patterns across the six treatments. (A) Numbers of increased and decreased responsive features in positive- and negative-ion modes across the seven primary contrasts. (B,C) UpSet plots showing exact intersections among responsive features in positive- and negative-ion modes, respectively. Bars indicate exact intersection sizes, and connected dots identify the contrasts contributing to each intersection. (D) Row-standardized abundance profiles of representative QC-stable features across biological replicates of the six treatments; colors represent row-wise Z scores. Side annotations indicate feature metadata used to classify the displayed features. S, unselected laboratory line; T, α-solanine-selected line; AD, normal artificial diet; ADS, α-solanine-containing artificial diet; PL, potato foliage; POS and NEG, positive- and negative-ion modes, respectively.

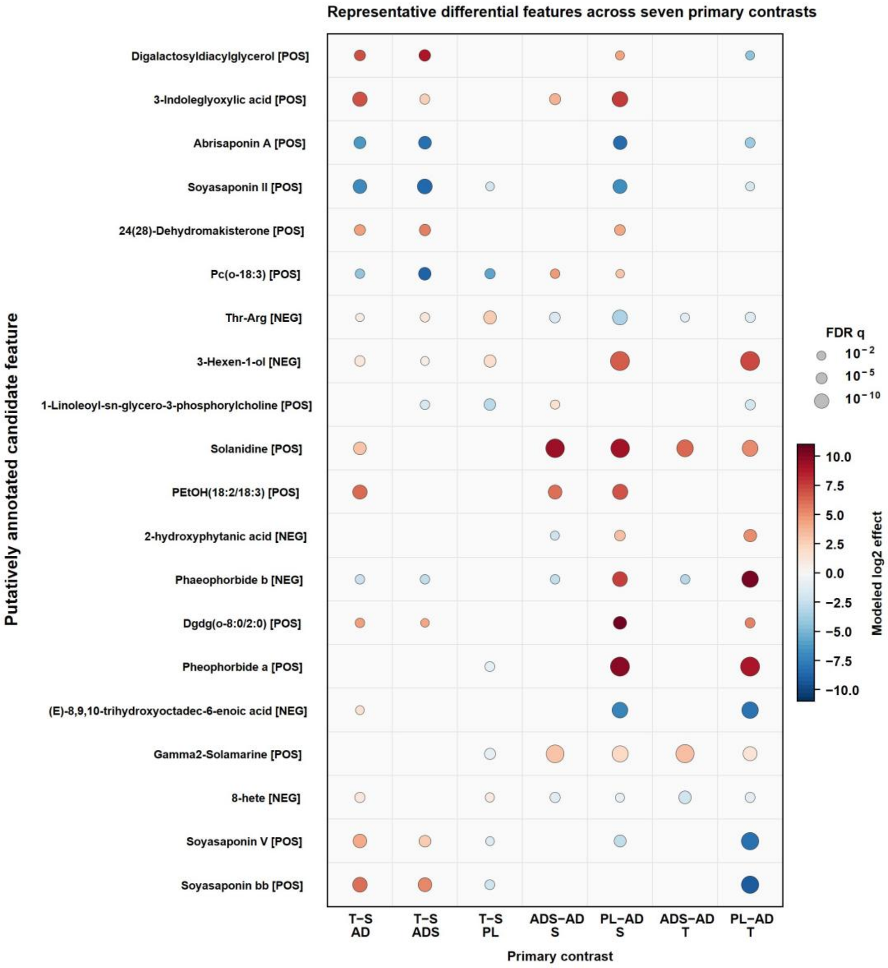

Figure S7. Representative differentially abundant candidate metabolic features across the seven primary contrasts. Color denotes the modeled $\log_2$ effect, and symbol size represents $-\log_{10}$(FDR q). Each row represents one putatively annotated candidate feature, and blank cells indicate contrasts in which that feature was not included in the representative significant-feature set. The feature set is identical to that reported in Table S2. S, unselected laboratory line; T, α-solanine-selected line; AD, normal artificial diet; ADS, α-solanine-containing artificial diet; PL, potato foliage.

Table S1. Primer sequences used for RT-qPCR analysis.

Gene	Forward primer (5′–3′)	Reverse primer (5′–3′)
SfMaf	GCGACCAGATAGTCCGAATGA	ACTCCTGGCTCTTCTCAGTCT
SfCYP9A32	CCAAGAACAACGGGAAGCTTG	CACACTCTGTCCATGAACGGA
SfCYP6AE44	TTTGGAGGTAAAGCAGTCCCC	GAGGTCCACAAAGTCGTTCCT
SfCPR	TGCCGGTTGTAAAGTCCTCTC	GTGCGCTGTCTGACATTTTGT
SfGSTD1	TACTACCTCCCACCTTCTCCG	ACTCCGGTGTCATGTGATGTC
SfABCC4	TTCTACGGCCTGCGGATATTC	GAGAGAATACCGGACTGCGAG
SfABCG1	CAGGAGCGTCAGGAACAATCT	AGGTCGGCAGCTATCATCATG
SfCAT	CTTCCGTGTGTCTGTCTCCAA	TTGGGGAAATAGTTGGGGCAA
SfCYP6B50	TCGCCTACACTGTTGATTCGT	GTCGGCGTGGAAAAGATTGTT
SfUGT40L8	CTGCCCAGGTGCTAATTCTCA	ACGGATGAGCGTTAATCAGCA
RPL10	GTCGTGCCAAGTTCAAGTTCC	CTCTTCACGCTCGTACTTGGT
RPS24	ACCTGGCTAAGAAGTTCGAGC	TACCACGCACCTTCTTCATCC

Table S2. Representative differentially abundant candidate metabolic features across the seven primary contrasts.

Feature names denote putative annotations from the processed LC–MS workflow. S, unselected laboratory line; T, α-solanine-selected line; AD, normal artificial diet; ADS, α-solanine-containing artificial diet; PL, potato foliage; POS and NEG, positive- and negative-ion modes, respectively.

Contrast	Direction	Putative annotation	m/z	RT (min)	Ion mode	log_2 effect	FDR q	QC RSD (%)
T−S | AD	Increased	Digalactosyldiacylglycerol	921.5940	22.34	POS	7.10	4.42×10^{-5}	3.0
T−S | AD	Increased	3-Indoleglyoxylic acid	212.0311	13.03	POS	7.01	3.84×10^{-11}	4.3
T−S | AD	Decreased	Abrisaponin A	943.5251	18.95	POS	-6.29	1.25×10^{-6}	17.2
T−S | AD	Decreased	Soyasaponin II	913.5161	19.35	POS	-6.93	5.68×10^{-10}	11.9
T−S | ADS	Increased	Digalactosyldiacylglycerol	921.5940	22.34	POS	8.87	4.55×10^{-6}	3.0
T−S | ADS	Increased	24(28)-Dehydromakisterone	531.2766	21.28	POS	5.54	5.54×10^{-6}	1.7
T−S | ADS	Decreased	Soyasaponin II	913.5161	19.35	POS	-8.60	1.71×10^{-11}	11.9
T−S | ADS	Decreased	Pc(o-18:3)	1035.6346	21.55	POS	-8.87	2.48×10^{-7}	11.8
T−S | PL	Increased	Thr-Arg	320.1577	15.00	NEG	2.77	1.96×10^{-8}	5.6
T−S | PL	Increased	3-Hexen-1-ol	135.0588	9.18	NEG	1.84	5.20×10^{-7}	6.8
T−S | PL	Decreased	1-Linoleoyl-sn-glycero-3-phosphorylcholine	520.3364	27.65	POS	-2.96	5.53×10^{-6}	4.0
T−S | PL	Decreased	Pc(o-18:3)	1035.6346	21.55	POS	-5.77	1.79×10^{-4}	11.8
ADS−AD | S	Increased	Solanidine	398.3426	14.54	POS	9.45	3.27×10^{-19}	0.9
ADS−AD | S	Increased	PEtOH(18:2/18:3)	740.5245	27.67	POS	6.08	8.99×10^{-10}	13.7
ADS−AD | S	Decreased	2-hydroxyphytanic acid	373.2987	22.89	NEG	-2.25	0.01	5.4
ADS−AD | S	Decreased	Phaeophorbide b	605.2388	24.03	NEG	-2.63	0.002	6.2
PL−AD | S	Increased	Dgdg(o-8:0/2:0)	593.2780	24.80	POS	10.62	1.64×10^{-8}	3.1
PL−AD | S	Increased	Pheophorbide a	1185.5396	24.80	POS	9.97	1.02×10^{-26}	7.2
PL−AD | S	Decreased	(E)-8,9,10-trihydroxyoctadec-6-enoic acid	659.4679	17.69	NEG	-7.22	2.68×10^{-13}	5.2
PL−AD | S	Decreased	Abrisaponin A	943.5251	18.95	POS	-8.44	3.06×10^{-9}	17.2
ADS−AD | T	Increased	Solanidine	398.3426	14.54	POS	6.36	8.10×10^{-15}	0.9
ADS−AD | T	Increased	Gamma2-Solamarine	722.4462	14.54	POS	3.32	4.14×10^{-18}	2.4
ADS−AD | T	Decreased	8-hete	355.2052	21.53	NEG	-2.14	1.44×10^{-7}	11.7
ADS−AD | T	Decreased	Phaeophorbide b	605.2388	24.03	NEG	-3.05	0.002	6.2
PL−AD | T	Increased	Phaeophorbide b	605.2388	24.03	NEG	10.40	5.03×10^{-15}	6.2
PL−AD | T	Increased	Pheophorbide a	1185.5396	24.80	POS	9.02	1.29×10^{-25}	7.2
PL−AD | T	Decreased	Soyasaponin V	959.5219	18.89	POS	-8.21	5.62×10^{-17}	3.4
PL−AD | T	Decreased	Soyasaponin bb	943.5279	19.17	POS	-9.19	2.43×10^{-16}	2.5

Table S3. Traditional cohort fertility-table parameters for treatments A–F.

Values are biological-replicate means ± SEM. At age x, m_x denotes age-specific egg production per surviving cohort individual, and R_0 denotes cumulative egg output per initial cohort individual. T, mean generation time; r_m, intrinsic rate of increase; λ, finite rate of increase.

Treatment	R_0 (cumulative egg output per initial cohort individual)	SEM	T (d)	SEM	r_m (d^{-1})	SEM	λ (d^{-1})	SEM	Doubling (d)	SEM
A	240.27	60.43	31.31	0.59	0.1733	0.0069	1.1892	0.0083	4.01	0.16
B	216.68	41.20	52.67	2.04	0.1019	0.0063	1.1073	0.0069	6.86	0.45
C	40.30	13.57	42.56	2.29	0.0823	0.0063	1.0858	0.0068	8.53	0.70
D	282.09	70.21	36.12	0.20	0.1509	0.0077	1.1630	0.0090	4.66	0.25
E	228.93	46.11	35.88	0.38	0.1484	0.0066	1.1601	0.0076	4.72	0.21
F	137.82	36.40	39.04	0.41	0.1218	0.0062	1.1297	0.0070	5.76	0.29
